# Nodal Facilitates Differentiation of Fibroblasts to Cancer-Associated Fibroblasts that Support Tumor Growth in Melanoma and Colorectal Cancer

**DOI:** 10.3390/cells8060538

**Published:** 2019-06-04

**Authors:** Ziqian Li, Junjie Zhang, Jiawang Zhou, Linlin Lu, Hongsheng Wang, Ge Zhang, Guohui Wan, Shaohui Cai, Jun Du

**Affiliations:** 1 Department of Microbial and Biochemical Pharmacy, School of Pharmaceutical Sciences, Sun Yat-sen University, Guangzhou 510006, China; liziqian@mail2.sysu.edu.cn (Z.L.); zhangjj59@mail2.sysu.edu.cn (J.Z.); zhoujw37@mail2.sysu.edu.cn (J.Z.); lull5@mail2.sysu.edu.cn (L.L.); whongsh@mail.sysu.edu.cn (H.W.); zhangge@mail.sysu.edu.cn (G.Z.); wanguoh@mail.sysu.edu.cn (G.W.); 2 Department of Pharmacology, School of Pharmaceutical Sciences, Jinan University, Guangzhou 510632, China; csh5689@sina.com

**Keywords:** Nodal, CAFs, differentiation, tumor growth, melanoma, colorectal cancer

## Abstract

Fibroblasts become cancer-associated fibroblasts (CAFs) in the tumor microenvironment after activation by transforming growth factor-β (TGF-β) and are critically involved in cancer progression. However, it is unknown whether the TGF superfamily member Nodal, which is expressed in various tumors but not expressed in normal adult tissue, influences the fibroblast to CAF conversion. Here, we report that Nodal has a positive correlation with α-smooth muscle actin (α-SMA) in clinical melanoma and colorectal cancer (CRC) tissues. We show the Nodal converts normal fibroblasts to CAFs, together with Snail and TGF-β signaling pathway activation in fibroblasts. Activated CAFs promote cancer growth in vitro and tumor-bearing mouse models in vivo. These results demonstrate that intercellular crosstalk between cancer cells and fibroblasts is mediated by Nodal, which controls tumor growth, providing potential targets for the prevention and treatment of tumors.

## 1. Introduction

Melanoma and colorectal cancer (CRC) are extremely malignant tumors due to their rapid development [1,2]. Tumorigenesis is driven by the complex intercellular communication in the tumor microenvironment of different cells, including tumor cells and stromal cells [3,4]. Cancer-associated fibroblasts (CAFs), the most abundant stromal cells in the tumor microenvironment, have been consistently researched over the decades regarding their interaction with cancer cells [5,6,7]. Normal fibroblasts are quiescent and are activated during wound healing and by tumors, leading to identification by various markers such as α-smooth muscle actin (α-SMA, encoded by the ACTA2 gene) [8]. Activated fibroblasts play a critical role in the proliferation and metastasis of tumors and the key factor in the formation and development of metastasis lesions [9]. However, the exact mechanisms by which fibroblasts differentiate into CAFs via tumor cells are still being elucidated and are even more obscure in melanoma and CRC.

Transforming growth factor-β (TGF-β) signaling plays an important role in tumor suppression or promotion, depending on the stage of tumorigenesis [10]. TGF-β activates fibroblasts by inducing intracellular signaling evens, such as phosphorylation and the nuclear translocation of Smad2 [11]. Snail, a TGF-β target gene, is a major player in TGF-β-mediated tumor promotion during tumorigenesis [11]. One of the member of the TGF superfamily, Nodal, an embryonic morphogen, is not expressed in healthy adult tissues but emerges in numerous cancers and is correlated with tumorigenesis, development, invasion, and metastasis [12,13,14]. Various studies have pointed out that TGF-β induces fibroblast differentiation and the conversion of activated fibroblasts into CAFs [15]. In addition, TGF-β and activin A, another TGF superfamily member, are involved in macrophage polarization [16,17]. As a member of the TGF superfamily, Nodal has functions that are similar to those of TGF-β [18]. However, whether Nodal is also involved in fibroblast differentiation in the tumor microenvironment has not been described clearly in melanoma and CRC.

In our study, we sought to further clarify the involvement of Nodal in fibroblast differentiation. By analyzing the expression of Nodal and α-SMA in melanoma, CRC patient tissues, and the Cancer Genome Atlas Program (TCGA database), we found a positive correlation between Nodal and α-SMA. Moreover, we show that Nodal contributes to the conversion of fibroblasts to CAFs in vitro through α-SMA detection compared to normal fibroblasts and those treated with self-derived Nodal protein, exogenous recombinant Nodal protein, or tumor cell-derived Nodal protein in a process that involves Snail and the TGF-β signaling pathway. Meanwhile, fibroblasts activated by Nodal promote melanoma and CRC proliferation in vitro and in vivo. The bilateral interaction between cancer cells and fibroblasts in the tumor microenvironment illuminates a novel mechanism of tumor progression and offers new opportunities for potential therapeutic strategies targeting tumor growth.

## 2. Materials and Methods

### 2.1. Ethics Approval and Consent to Participate

Human melanoma and CRC tumor tissues were obtained from patients at the First Affiliated Hospital of Clinical Medicine of Guangdong Pharmaceutical University in Huizhou, China. The study was approved by the Ethical Committee of Pharmaceutical Sciences, Sun Yat-sen University, under the Chinese Ethical Regulations.

The 4–5-week-old female BALB/c-nude mice and BALB/c mice were provided by the Animal Experimental Center of Sun Yat-sen University (Guangzhou, China) and housed in the Laboratory Animal Center under specific pathogen-free conditions. The experimental handling and care procedures for the mice were approved by the Animal Experimentation Ethics Committee of Sun Yat-sen University (Guangzhou).

### 2.2. Tissue Samples and Histological Study

Immunohistochemistry (IHC) was performed to measure the expression of Nodal, α-SMA, and proliferating cell nuclear antigen (PCNA). In brief, fresh tumor tissues were embedded in paraffin after fixation in formalin. Then, 4-μm sections were cut, deparaffinized, and hydrated. Next, 3% H_2_O_2_ was used to block endogenous peroxidase activity for 20 min. The slides were blocked with normal goat serum at 37 °C for 45 min after high-pressure antigen retrieval in citric acid buffer (pH = 6.0) for 10 min. Slides were incubated with primary antibodies overnight at 4 °C. After three washes in 0.1% Tween-20 phosphate buffer solution (PBST), the slides were incubated with secondary antibodies for 2 h at room temperature and then washed again. Finally, the slides were counterstained with hematoxylin after staining with a diaminobenzidine kit. PBS was used in place of the primary antibodies for negative controls. Microscopy was used to observe the stained sections. The following antibodies were used for IHC and western blotting: mouse monoclonal anti-Nodal (1:200, ab55676, Abcam, Cambridge, MA, USA), mouse monoclonal anti-α-SMA (1:200, BM0002, BOSTER Biological Technology, Pleasanton, CA, USA), rabbit polyclonal anti-PCNA (1:200, BS1289, Bioworld Technology, St. Louis Park, MN, USA), horseradish peroxidase (HRP)-conjugated anti-mouse and HRP-conjugated anti-rabbit secondary antibodies (1:200, Bioworld).

### 2.3. Evaluation of IHC Staining

Three independent scorers (Jiawang Zhou, Junjie Zhang, and Ziqian Li) observed the stained slides and recorded the scores by assessing (a) the proportion of positively stained cells (0, <5%; 1, 6–25%; 2, 26–50%; 3, 51–75%; 4, 76–100%) and (b) the intensity of staining (0, negative; 1, weak staining; 2, medium staining; 3, strong staining). The score was calculated by a × b.

### 2.4. Cell Lines and Culture

Mouse fibroblast (3T3), mouse melanoma (B16), mouse colorectal cancer (CT26), human melanoma (A375), and human skin fibroblasts (HSF) cell lines were purchased from the Institute of Biochemistry and Cell Biology, Chinese Academy of Sciences (Shanghai, China). B16-Nodal, B16-shNodal, CT26-Nodal, CT26-shNodal, A375-Nodal, and A375-shNodal stable cell lines were generated based on our previous methods [19]. To generate Nodal stable overexpression/silencing cells, the wild-type cells were transfected with pLd-Nodal/pGFP-shNodal vectors via liposome-mediated transfection. The transfected cells were selected with G418 (800 μg/mL)/puromycin (7 μg/mL) for 2 weeks. The survived cells were passed and seeded into a 96-well plate for the formation of cell clones and further expansion. 3T3, B16, and CT26 were maintained in Dulbecco modified Eagle’s medium (DMEM; GIBCO, Invitrogen, Grand Island, NY, UK) while A375 and HSF were cultured in RPMI-1640 (GIBCO) supplemented with 10% fetal bovine serum (FBS) and 1% penicillin/streptomycin (Invitrogen, Grand Island, NY, UK) at 37 °C under a humidified 5% CO_2_ atmosphere.

### 2.5. Western Blotting

Cells were harvested and rinsed with PBS at the indicated times. The total protein was prepared with Radio Immunoprecipitation Assay (RIPA) buffer (Beyotime Institute of Biotechnology, Jiangsu, China), containing 1 mM phenylmethanesulfonyl fluoride (PMSF) and centrifuged at 12,000 rpm/min for 20 min at 4 °C. Protein samples were quantified using a BCA Protein Assay Kit (Beyotime Biotechnology) and electrophoresed using 10% polyacrylamide gels and transferred onto polyvinylidene fluoride (PVDF) membranes (Millipore, Billerica, MA, USA). The members were probed with primary antibodies (final dilution, 1:1000, rabbit polyclonal, provided by Cell Signaling Technology, Beverly, MA, USA) overnight at 4 °C after blocking with 5% non-fat dried milk for 120 min at room temperature. GAPDH and α-tubulin were used as the loading controls. After washing with PBST three times, the membranes were incubated with secondary antibodies labeled with HRP (final dilution, 1:5000) and then washed. The signals were observed by Chemiluminescence Reagent (Life Science, Inc., Boston, MA, USA) in a Tanon 5200 Multi instrument (Shanghai, China).

For densitometric analyses, protein bands on the blots were measured by ImageJ software.

### 2.6. Real-Time PCR

The real-time PCR assays were performed as previously described [20]. The primers used in each reaction were as follows: Nodal (NM_013611.4), forward 5’-TAC ATG TTG AGC CTC TAC CGA GAC C-3´ and reverse 5´-AAA CGT GAA AGT CCA GTT CTG TCC-3´; GAPDH (NM_008084.2), forward 5´-TGT GTC CGT CGT GGA TCT GA-3´ and reverse 5´-TTG CTG TTG AAG TCG CAG GAG-3´. The threshold cycle (CT) values of Nodal were normalized by the values of housekeeping gene GAPDH. The relative fold changes in mRNA expression level were calculated with the comparative CT method.

### 2.7. Cell Growth Analysis and Co-Culture Assay

The cell growth of B16 and CT26 with fibroblasts activated by Nodal was evaluated by Cell Counting Kit-8 (CCK-8; Dojindo, Kumamoto, Japan) according to previously described procedures [20]. The co-culture assay was established using 6-well Millicell Hanging Cell Culture Inserts (Millicell, Sigma, St. Louis, MO, USA) with a 0.8-μm pore size. In brief, 5 × 10^4^ tumor cells were seeded on the bottom of the 6-well culture plate and 10^5^ fibroblasts treated with 600 ng/mL Nodal protein for 24 h were plated on the Transwell membranes (Millicell, Sigma, St. Louis, MO, USA). The cells were incubated for 48 h and the tumor cells were collected for further experiments.

### 2.8. Xenograft Tumor

In total, for the simple subcutaneous transplanted model, Nodal overexpression and silencing cells (5 × 10^6^ per mouse, *n* = 5 for each group) were diluted in 200 μL of normal saline and subcutaneously injected into mice. For the mixed subcutaneous transplanted model, Nodal overexpression and silencing cells (5 × 10^6^ per mouse, *n* = 5 for each group) were mixed with 3T3 cells at a 1:2 ratio in 200 μL of normal saline and injected into nude mice subcutaneously under the right shoulder. The BALB/c mice were inoculated subcutaneously with CT26 and the immunodeficient mice were used for B16 cells. The day of tumor inoculation was designated as day 1. Until the tumor volumes grew to approximately 100 mm^3^ (7 days), the subcutaneous tumor volumes were measured every other day by a caliper. The tumor volume calculation formula was as follows: volume = 0.5 × length × width × width.

### 2.9. Preparation of Protein and RNA from the Xenograft Tissue

The half xenograft tumor tissues were collected and dissected into 3–4 mm pieces with scissors in a saline salt solution. For protein, tissues were placed in 1.5-mL microcentrifuge tubes with RIPA (100 mg tissue in 1 mL RIPA), containing 1 mM PMSF. For RNA, 100-mg tissues were placed in 1.5-mL microcentrifuge tubes with 1 mL TRIZOL (Thermo Fisher Scientific). The handheld homogenizer was used to disrupt tissues at 4 °C. Then the protein and RNA isolation protocols were started as described in Section 2.5 and Section 2.6.

### 2.10. Immunofluorescence Assay

3T3 and HSF cells were grown on a coverslip in 6-well plates. After they were treated with 600 ng/mL recombinant Nodal protein or blocked by Nodal antibody (10 μg/mL) for 48 h, cells were fixed in 4% paraformaldehyde for 30 min, blocked with normal goat serum, and then incubated with α-SMA antibody (final dilution, 1:200) at 4 °C overnight. After being incubated with fluorescein isothiocyanate (FITC)-conjugated goat anti-mouse antibody and having their nuclear contents stained with diaminophenylindole (DAPI), cells were analyzed by immunofluorescence microscopy.

### 2.11. Statistical Analysis

In the mouse studies, five biological replicates were utilized, whereas there were three biological replicates in all other studies. All statistical analyses were performed using IBM SPSS Statistics ver. 20 (IBM Corp., Armonk, NY, USA) for Windows. In all cases, a *p*-value of <0.05 was considered statistically significant. The unpaired two-tailed Student’s *t* test was used to analyze two groups and one-way ANOVA was used for multiple comparisons.

## 3. Results

### 3.1. Correlation of α-SMA and Nodal Expression in Human Melanoma and CRC Tissues Indicates Nodal Plays a Role in Fibroblasts

**Figure 1:**
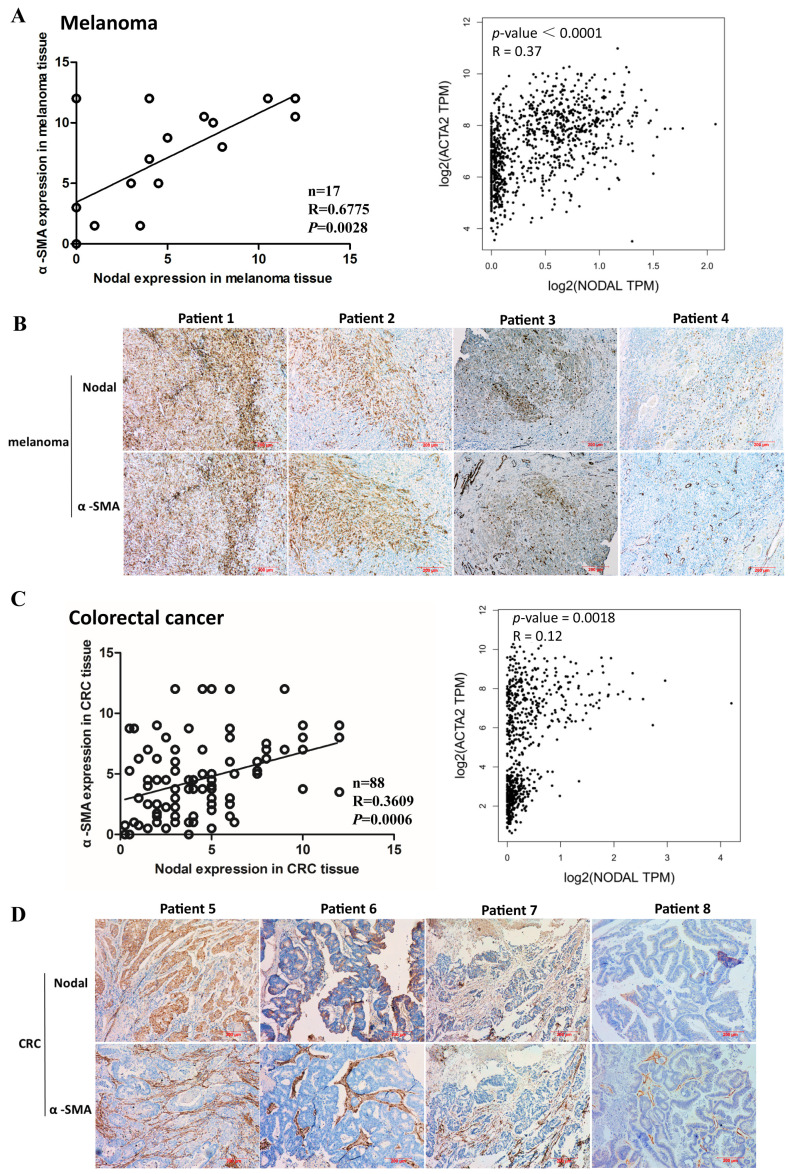
Correlation of α-smooth muscle actin (α-SMA) and Nodal expression in human melanoma and colorectal cancer (CRC) tissues. (**A**) The levels of α-SMA and Nodal expression in human melanoma were detected by immunohistochemistry (IHC) and evaluated (left). Correlation between α-SMA and Nodal mRNA expression in melanoma cancer tissues from the Cancer Genome Atlas Program (TCGA database; right). (**B**) Representative immunohistochemical images of α-SMA and Nodal expression in human melanoma tissues. (**C**) The levels of α-SMA and Nodal expression in human CRC were detected by IHC and evaluated (left). Correlation between α-SMA and Nodal mRNA expression in CRC tissues from TCGA database (right). (**D**) Representative immunohistochemical images of α-SMA and Nodal expression in human CRC tissues.

CAFs have complex interactions with cancer cells. Previous studies observed that Nodal, a member of the TGF superfamily, was aberrantly expressed in many malignant tumors [12]. In addition, fibroblasts were activated by growth factors such as TGF-β, chemokines, and cytokines [21]. Hence, we hypothesized that Nodal was correlated with CAFs. To confirm this correlation, we performed immunohistochemistry to examine Nodal and α-SMA expression to identify the most effective CAF marker in 17 melanoma and 88 CRC cases. Based on the scoring criteria described in the methods section, the Nodal and α-SMA expression scores are shown in Tables S1 and S2. The correlation analysis (protein expression) and TCGA data (RNA expression) showed that expression of Nodal and α-SMA was positively correlated (Figure 1A,C). IHC results showed that Nodal expression was positively correlated with α-SMA expression in tumor tissues (Figure 1B,D), indicating that Nodal may play an important role in CAFs.

### 3.2. Nodal Facilitates the Differentiation of Fibroblasts into CAFs

**Figure 2:**
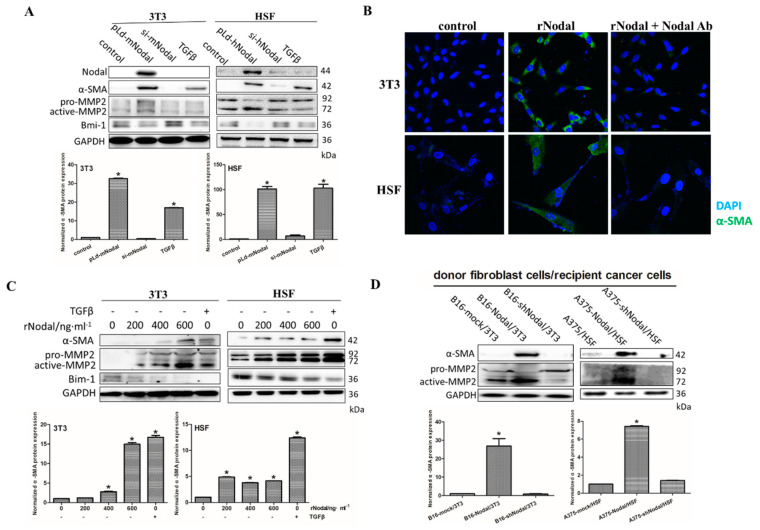
Nodal facilitates the differentiation of fibroblasts to CAFs. (**A**) After transfection with pLd-Nodal or siNodal or the presence of 10 ng/mL transforming growth factor-β (TGF-β), protein expression of Nodal, α-SMA, MMP2, and Bmi-1 in 3T3 and human skin fibroblasts (HSF) cell lines was measured by western blot (up) and quantitatively analyzed (below). (**B**) α-SMA immunofluorescence staining of 3T3 and HSF cells treated with recombinant Nodal protein or blocked by 10 μg/mL Nodal antibody (Abcam, Cambridge, MA, USA) are presented. (**C**) The protein expression of α-SMA, MMP2, and Bmi-1 in 3T3 and HSF cells treated with 200, 400, and 600 ng/mL recombinant Nodal protein or TGF-β was detected by western blot (up) and quantitatively analyzed (below). (**D**) α-SMA and MMP2 expression of 3T3 and HSF cells co-cultured with B16, B16-Nodal, B16-shNodal, A375, A375-Nodal, and A375-shNodal were analyzed by western blot (up) and quantitatively analyzed (below). * *p* < 0.05.

Many factors derived by activated fibroblasts, such as MMP2 and fibroblast growth factor 1 (FGF1), can promote profound proliferation of cancer cells [9]. Additionally, Bmi-1 is a polycomb group gene that inhibits senescence and enhances immunomodulatory properties [22]. The decreased expression of Bmi-1 indicates the differentiation of cells. To further identify the role of Nodal in fibroblasts, we characterized phenotypic changes in the normal mouse fibroblast 3T3 cell line and the normal human skin fibroblast (HSF) cell line after Nodal treatment by western blotting and immunofluorescence. Compared with the control group, the expression of α-SMA and active-MMP2 in 3T3 and HSF was increased, showing that the fibroblasts were activated and the stem cell phenotype was decreased after Nodal overexpression (Figure 2A). In addition, after treatment with exogenous recombinant Nodal protein, 3T3 and HSF displayed CAFs properties, which was reversed by the neutralizing Nodal antibody (Figure 2B). The normal fibroblasts were activated by Nodal in a concentration-dependent manner (Figure 2C). Furthermore, 3T3 and HSF differentiated into CAFs after co-culture with Nodal-overexpressing cancer cells (Figure 2D), indicating that Nodal facilitated the differentiation of normal fibroblasts into CAFs.

### 3.3. Snail Contributes to Nodal-Induced Fibroblast Differentiation via TGF-β Signaling In Vitro

**Figure 3:**
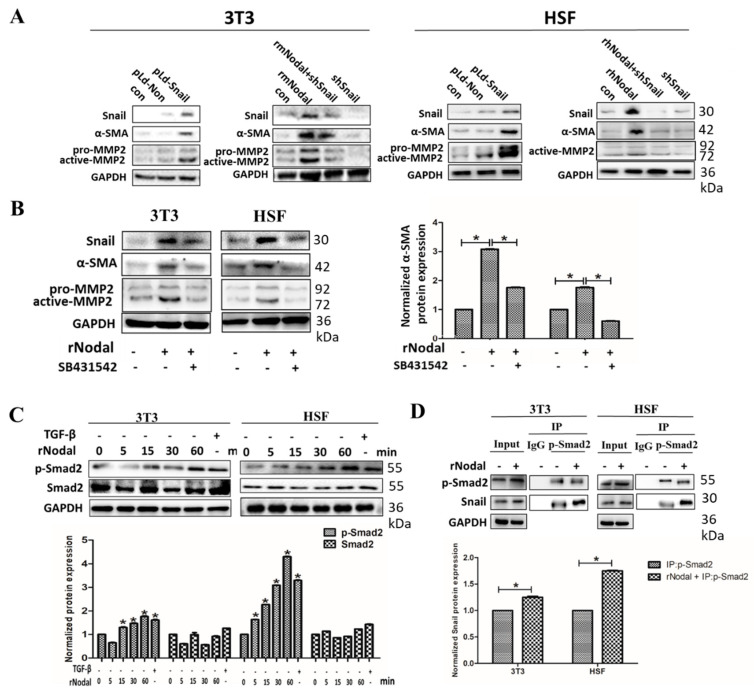
Snail contributes to fibroblasts differentiation induced by Nodal via TGF-β signaling pathways. (**A**) 3T3 and HSF cells were transfected with pLd-Snail or treated with Nodal protein in the absence or presence of the shSnail plasmid, and the expression of Snail, α-SMA, and MMP2 was determined by western blot. (**B**) 3T3 and HSF were pretreated with or without SB431542 (20 μM) for 2 h and then treated with Nodal protein (600 ng/mL) for 48 h. The expression of Snail, α-SMA, and MMP2 was analyzed by western blot (left) and the expression of α-SMA was quantitatively analyzed (right). (**C**) 3T3 and HSF cells were treated with Nodal protein for 5 min, 15 min, 30 min, and 1 h or TGF-β for 30 min. The total and phosphorylation level of Smad2, a key protein of the TGF-β signaling pathway, was detected by western blot (up) and quantitatively analyzed (below). (**D**) 3T3 and HSF cells were treated with Nodal protein (600 ng/mL) for 12 h, and then Snail was immunoprecipitated from equal amounts of lysates and the associated Snail and phosphorylated-Smad2 was analyzed by western blot (up) and the expression of Snail was quantitatively analyzed (below). * *p* < 0.05.

We next explored how Nodal activates fibroblasts. Previous studies have reported that TGF-β signaling dominates fibroblast differentiation into myofibroblasts [23,24,25]. As members of the TGF superfamily, Nodal and TGF-β have similar characteristics. Snail has been described as a TGF-β target, and our previous studies also showed that Snail participated in Nodal-induced epithelial-to-mesenchymal transition processes [19]. Additionally, Snail-overexpressing fibroblasts displayed CAF properties in our previous research [20]. Therefore, we hypothesized that Snail participates in the fibroblast activation induced by Nodal through the TGF-β signaling pathway. Similar results were observed in 3T3 and HSF cells activated by Nodal, as the expression of Snail and differentiation was inhibited after silencing Snail expression (Figure 3A).

Alternatively, the induction of mothers against decapentaplegic homolog (Smad2) signaling by the TGF-β pathway may be the dominant mechanism underlying the transformation induced by Nodal. Thus, we next examined total Smad2 and phosphorylated-Smad2 by western blot. Furthermore, fibroblast differentiation was inhibited by SB431542, a TGF-β receptor inhibitor (Figure 3B). As shown in Figure 3C, Smad2 phosphorylation was upregulated in a time-dependent manner in the Nodal-treated groups compared to the control groups, implying that Smad2 pathway activation was essential for the fibroblast differentiation induced by Nodal. Moreover, Snail was associated with p-Smad2, and the association was increased in cells treated with Nodal (Figure 3D). These data suggest that Nodal induces fibroblast transdifferentiation into CAFs through the Smad pathway with Snail acting as an important participator in this process.

### 3.4. Fibroblasts Activated by Nodal Support Tumor Growth in Melanoma and CRC In Vitro and In Vivo

**Figure 4:**
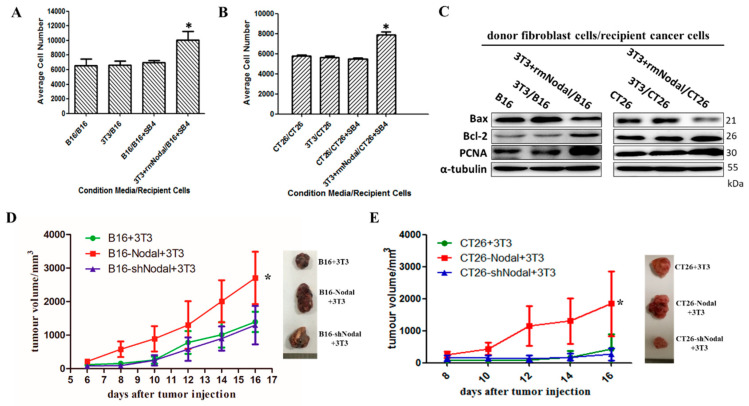
Fibroblasts activated by Nodal support tumor growth in melanoma and CRC. (**A**) B16 and (**B**) CT26 cells in the presence of SB431542 were grown in 3T3-conditioned media or 3T3-Nodal-treated-conditioned media without FBS for 48 h and the total number of cells was counted by Cell Counting Kit 8 (*p < 0.05). (**C**) The level of Bax, Bcl-2, and PCNA proteins from B16 or CT26 alone or co-cultured with 3T3 pretreated with Nodal protein were analyzed by western blotting. (**D**) Tumor growth curves of B16 + 3T3, B16-Nodal + 3T3, and B16-shNodal + 3T3 groups and the representative images of tumors are presented. (**E**) Tumor growth curves of CT26 + 3T3, CT26-Nodal + 3T3, and CT26-shNodal + 3T3 groups and the representative images of tumors are presented. * *p* < 0.05.

CAFs are essential for the progression of many tumors [3,26]. To determine whether fibroblasts activated by Nodal contribute to the promotion of tumor growth, we performed a set of experiments in vitro and in vivo. First, to confirm the effects mediated by tumor cells on fibroblasts are related to Nodal rather than TGF-β in our experiments, the expression of Nodal and TGF-β in B16, B16-Nodal, B16-shNodal, CT26, CT26-Nodal, and CT26-shNodal were checked by western blot. As shown in Figure S1, TGF-β expression in those cells almost did not change, while Nodal expression was significantly increased in Nodal-overexpression cells and decreased in Nodal-silencing cells. In order to verify whether Nodal affects the tumor cells growth in vitro and in vivo, recombinant Nodal protein was used to treat tumor cells and B16, B16-Nodal, B16-shNodal, CT26, CT26-Nodal, and CT26-shNodal cells were used to establish xenograft tumor models. Results showed that the proliferation of cells did not change after treatment with Nodal (Figure S2), which indicated that Nodal did not affect tumor proliferation. Then, B16 and CT26 tumor cells were grown in conditioned media from 3T3 fibroblasts pretreated with Nodal. As shown in Figure 4A,B, tumor cells exhibited enhanced growth compared with control groups. SB432542 is a selective inhibitor of Nodal signaling that blocks the downstream p-Smad2 signaling cascade. As a result, SB431542 treatment was used to block Nodal function. Moreover, PCNA is known as an index to evaluate cell proliferation status and cell apoptosis is stimulated by Bax while inhibited by Bcl-2 [27,28]. We found that PCNA and Bcl-2 were increased in tumor cells co-cultured with activated 3T3, while the expression of Bax was decreased (Figure 4C). The data indicated that co-culture with activated 3T3 cell lines increased B16 and CT26 proliferation. Our previous reports had proved that Nodal mainly affect cell invasion, but not proliferation, in vivo. In addition, Nodal-overexpressing cell lines were mixed with 3T3 in vivo (Figure 4D,E). The xenograft model groups established with Nodal-silenced or normal CT26 tumor cells with 3T3 cells grew more slowly. Of note, the B16-Nodal + 3T3 and CT26 + 3T3 groups showed significantly increased proliferation.

**Figure 5:**
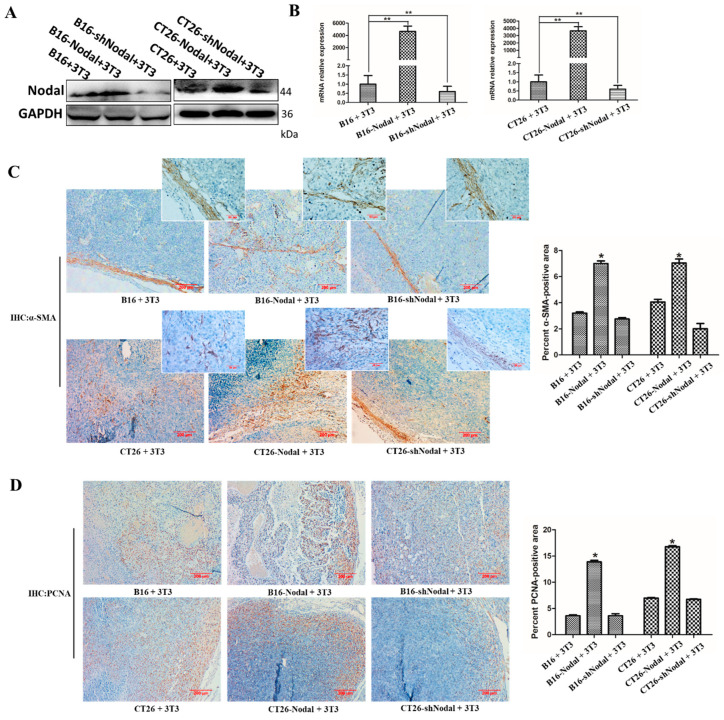
The distribution of CAFs and the expression of PCNA in the tumor tissue sections. (**A**) Western blotting of Nodal and TGF-β in xenograft tissues. GAPDH served as the loading control. (**B)** The mRNA level of Nodal in xenograft tissues was detected by real-time PCR. (**C**) Representative images of α-SMA-stained paraffin sections of B16 and CT26 tumor tissues were shown (×100; left) and quantitatively analyzed by ImageJ (right). The figures in the top right corner were enlarged (×400). (**D**) Representative images of PCNA staining in paraffin sections were shown (×100; left) and quantitatively analyzed by ImageJ (right). * *p* < 0.05, ** *p* < 0.01.

To confirm the Nodal expression in xenograft tumor tissues, we collected half tumor tissues and isolated protein and RNA. Our data showed that the expression of Nodal in the B16-Nodal+3T3 and CT26 + 3T3 groups was higher than the normal groups, and that the expression in the silencing groups was lower than in the normal groups (Figure 5A,B). Cancer cell numbers are often not correlated with tumor volume in vivo. To evaluate more definitively the function of fibroblasts activated by Nodal, we collected the tumor tissues and detected the quantity of CAFs and the expression of PCNA by IHC. Fibroblasts in tumor stroma usually express α-SMA. The α-SMA staining showed that more CAFs were localized in the groups containing Nodal-overexpressing tumor cells combined with 3T3 cells (Figure 5C). Furthermore, PCNA expression levels were increased in the groups containing Nodal-overexpressing tumor cells combined with 3T3 cells, which were different from the normal or Nodal-silenced tumor cells combined with 3T3 groups (Figure 5D). In summary, our results show that Nodal converts fibroblasts to CAFs to promote the tumor growth of melanoma and CRC.

## 4. Discussion

Fibroblasts, which exist in a quiescent state in tissues, are triggered to differentiate into myofibroblasts, also called CAFs, which disrupts tissue structures, including tumors [29]. The tumor microenvironment is orchestrated by intercellular communications through a dynamic system [30]. Accumulating evidence has revealed that CAFs, the major component of stroma in malignancies, play an important role in tumor proliferation and are potential targets for cancer therapy [5,31]. Therefore, there is a pressing need to identify the interaction between tumor cells and fibroblasts and to understand the functions and mechanisms of fibroblasts. Here, using IHC staining of clinical samples and TCGA data from melanoma and CRC patients, we found that the expression of Nodal in tumor tissues was positively corrected with α-SMA, suggesting that Nodal may play a crucial role in fibroblasts. In this study, we first detected the Nodal and TGF-β expression in the stable tumor cells that we used in the following experiments. We found that the TGF-β expression in our stable cells did not change while Nodal expression changed significantly. As a result, we believe that the differential effect on fibroblasts is mainly due to the presence of Nodal rather than TGF-β. Then cells transfected with a Nodal-overexpressing plasmid, treated with recombinant Nodal protein, or co-cultured with Nodal-expressing tumor cells were used to stimulate normal 3T3 and HSF fibroblasts. We found that normal fibroblasts were activated via the expression of α-SMA. Moreover, recent studies reported that a subset of mesodermal-derived cells, mainly activated fibroblasts, expressed Snail, while the bulk of adult epithelial cells and fibroblasts did not [32]. This restricted expression implies that Snail provides fibroblasts with additional properties. Not surprisingly, we found that Snail could also convert fibroblasts into CAFs in our previous study [20]. More recently, Tillaux et al. showed that Snail was upregulated via the TGF-β-Smad signaling pathway in activated fibroblasts and CAFs [33]. Similarly, we found that Nodal converted fibroblasts to CAFs by activating Smad2 signaling in vitro. Combined with the immunoprecipitation (IP) analysis, we proved that p-Smad2 was associated with Snail in regulating the differentiation of fibroblasts. In our study, Nodal derived by tumor cells activated the Smad2 signaling in fibroblasts and the phosphorylated Smad2 was associated with Snail in promoting the activation of fibroblasts. However, the underlying reasons for the differential effects of Nodal on fibroblasts in vivo warrant further study.

Previous studies have shown that CAFs promote cancer progression, including cancer formation, proliferation, chemoresistance, and metastasis [34,35]. Our data reveal that fibroblasts activated by Nodal supported the growth of B16 and CT26 cells. Moreover, animal models established by tumor cells with fibroblasts in a 1:2 ratio demonstrated that Nodal facilitated the differentiation of fibroblasts to CAFs and further promoted tumor cell growth. Given that the reduction and increases in cancer cell number are often not associated with comparable decreases and increases in tumor volume [36], the expression of α-SMA and PCNA was detected by IHC staining. It is obvious that PCNA expression was positively correlated with α-SMA, and they were both upregulated in the Nodal-overexpression groups. CAFs play an important role in tumor growth by releasing growth factors, cytokines, metalloproteinases (MMPs), and microRNAs [37]. Nevertheless, the exact mechanism by which CAFs support tumor growth remains unknown.

## 5. Conclusions

In this context, our findings highlight the therapeutic potential target of Nodal, as it significantly converts normal fibroblasts to CAFs through Smad2/Snail pathway and thus promotes the proliferation of tumors, where the upregulation of Nodal is frequently observed [12]. Furthermore, given that no single signaling pathway in tumor cells exclusively stimulates fibroblasts in the tumor microenvironment, the targeting of Nodal as a therapeutic option to suppress tumor progression warrants further investigation.

## Supplementary Materials

The following are available online at https://www.mdpi.com/2073-4409/8/6/538/s1, Figure S1: Nodal expression in Nodal-overexpression or silencing stable cells, Figure S2: Nodal do not support tumor growth in melanoma and CRC, Table S1: The average of three scorers’ evaluation valuation for α-SMA and Nodal staining of 17 patients with melanoma, Table S2: The average of three scorers’ evaluation for α-SMA and Nodal staining of 88 patients with colorectal cancer.

## Abbreviations

CAFs: cancer-associated fibroblasts; TGF-β: transforming growth factor-β; α-SMA: α-smooth muscle actin; IHC: immunohistochemical; CRC: colorectal cancer; MMPs: metalloproteinases; PCNA: proliferating cell nuclear antigen; DMEM: Dulbecco modified Eagle’s medium; CCK-8: Cell Counting Kit-8; DAPI: diaminophenylindole; FGF1: fibroblast growth factor 1; HSF: human normal skin fibroblast
