# Correction: Li et al. Nodal Facilitates Differentiation of Fibroblasts to Cancer-Associated Fibroblasts that Support Tumor Growth in Melanoma and Colorectal Cancer. *Cells* 2019, *8*, 538

**DOI:** 10.3390/cells13232020

**Published:** 2024-12-06

**Authors:** Ziqian Li, Junjie Zhang, Jiawang Zhou, Linlin Lu, Hongsheng Wang, Ge Zhang, Guohui Wan, Shaohui Cai, Jun Du

**Affiliations:** 1 Department of Microbial and Biochemical Pharmacy, School of Pharmaceutical Sciences, Sun Yat-sen University, Guangzhou 510006, China; 2 Department of Pharmacology, School of Pharmaceutical Sciences, Jinan University, Guangzhou 510632, China

## Error in Figure

In the original publication, there was a mistake in Figure 1B as published. The immunohistochemistry image of α-SMA in Figure 1b for patient 4 in our article published in *Int. J. Cancer* [1] was inadvertently reused as the image for patient 3 in Figure 1B of our manuscript published in *Cells* [2]. The corrected Figure 1 is as appears below. The authors state that the scientific conclusions are unaffected. This correction was approved by the Academic Editor. The original publication has also been updated.

## Figures and Tables

**Figure 1:**
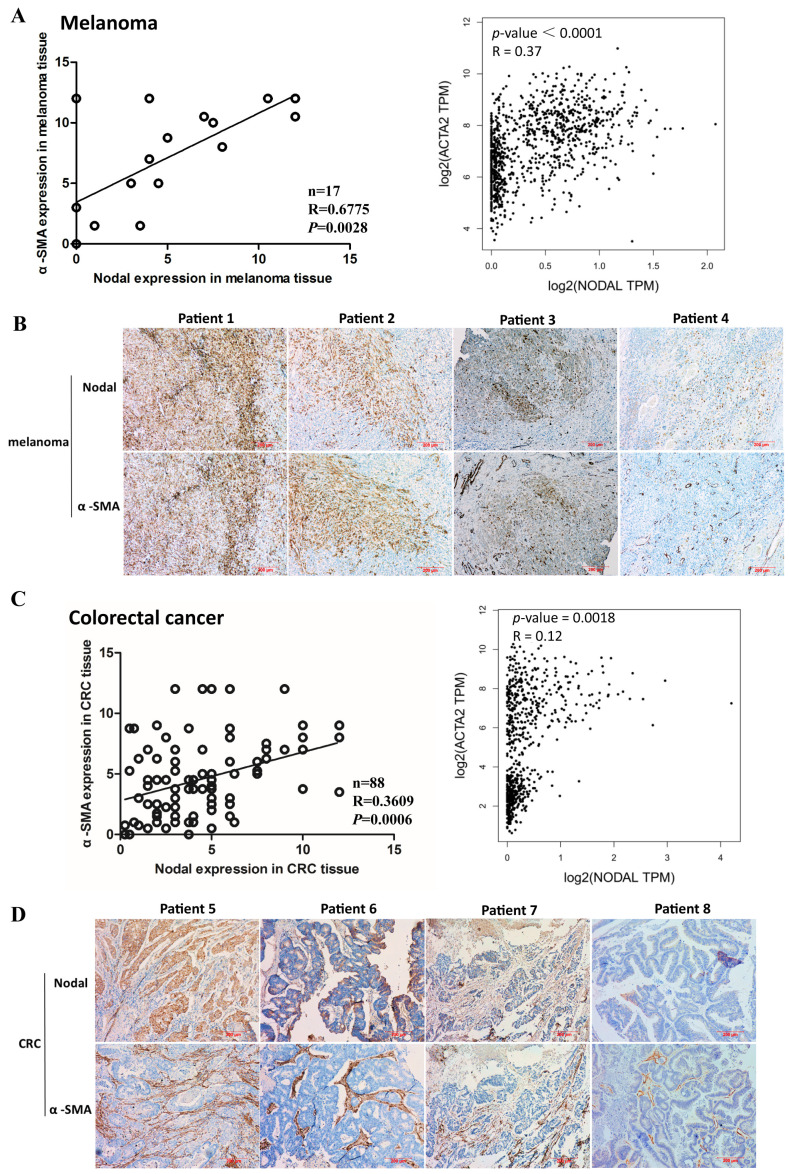
Correlation of α-smooth muscle actin (α-SMA) and Nodal expression in human melanoma and colorectal cancer (CRC) tissues. (**A**) The levels of α-SMA and Nodal expression in human melanoma were detected by immunohistochemistry (IHC) and evaluated (left). Correlation between α-SMA and Nodal mRNA expression in melanoma cancer tissues from the Cancer Genome Atlas Program (TCGA database; right). (**B**) Representative immunohistochemical images of α-SMA and Nodal expression in human melanoma tissues. (**C**) The levels of α-SMA and Nodal expression in human CRC were detected by IHC and evaluated (left). Correlation between α-SMA and Nodal mRNA expression in CRC tissues from TCGA database (right). (**D**) Representative immunohistochemical images of α-SMA and Nodal expression in human CRC tissues.
